# Symmetric vaccine efficacy

**DOI:** 10.1093/biomtc/ujag141

**Published:** 2026-08-11

**Authors:** Lucy D’Agostino McGowan, Sarah C Lotspeich, Michael G Hudgens

**Affiliations:** Department of Statistical Sciences, Wake Forest University, Winston-Salem, NC 27106, United States; Department of Statistical Sciences, Wake Forest University, Winston-Salem, NC 27106, United States; Department of Biostatistics University of North Carolina at Chapel Hill, Chapel Hill, NC 27599, United States

**Keywords:** communication, infectious disease, public health, relative risk, symmetric metrics, vaccination

## Abstract

Traditional measures of vaccine efficacy (VE) are inherently asymmetric, constrained above by 1 but unbounded below. As a result, VE estimates and corresponding confidence intervals (CIs) can extend far below zero, making interpretation difficult and potentially obscuring whether the apparent effect reflects true harm or simply statistical uncertainty. The proposed symmetric vaccine efficacy (SVE) is a bounded and interpretable alternative to VE that maintains desirable statistical properties while resolving these asymmetries. SVE is defined as a symmetric transformation of infection risks, with possible values within $[-1, 1]$, providing a common scale for both beneficial and harmful vaccine effects. This paper describes the relationship between SVE and traditional VE, considers inference about SVE, and illustrates the utility of the proposed measure by reanalyzing data from a randomized trial of a candidate HIV vaccine. Open-source tools for computing estimates of SVE and corresponding CIs are available in R through the sve package.

## 1 Introduction

Vaccines are one of the most effective public health interventions for preventing infectious diseases. Quantifying vaccine effects accurately and providing interpretable estimates is critical for regulatory decisions, public health policy, and individual risk communication. The most commonly used measure of vaccine performance is vaccine efficacy (VE), typically defined as one minus the relative risk of infection if vaccinated versus not vaccinated (Halloran et al., 1991; World Health Organization, 2025). Throughout this paper, the term *infection* is used to describe the outcome of interest for consistency and clarity; however, the framework applies equally to any binary outcome commonly measured in vaccine trials (eg, symptomatic disease, hospitalization, or death).

The parameter space for traditional VE is inherently asymmetric, with VE bounded above by 1 (complete protection afforded by vaccination) but unbounded below. Consequently, VE estimates and corresponding confidence intervals (CIs) can take on extreme negative values when the vaccine group experiences higher infection rates or the numbers of individuals infected in both groups are small. Such extremes have been observed in certain subgroups of HIV vaccine trials (rgp120 HIV Vaccine Study Group, 2005) and analyses of COVID-19 vaccine trials (Bodner et al., 2023; Buchan et al., 2022; Hansen et al., 2021). Asymmetry in VE complicates interpretation, particularly when communicating results in settings with small numbers of infections where CIs can be wide.

Symmetrization of effect measures has been proposed in other contexts to improve interpretability by placing beneficial and harmful treatment effects on a common scale. For example, Berry and Ayers (2006) introduced the *symmetrized percent change* for treatment comparisons from baseline, demonstrating improved interpretability without loss of statistical efficiency. More broadly, effect measures that switch form depending on the direction of the treatment effect have appeared in the statistical literature. For example, van der Laan et al. (2007) proposed the switch causal relative risk for binary outcomes in the context of randomized trials with noncompliance, and Baker and Jackson (2018) proposed the generalized relative risk reduction in the context of meta-analyses. Huitfeldt et al. (2021) showed that such effect measures have desirable stability properties. This paper considers a similar approach to vaccine evaluation, defining a symmetric vaccine efficacy (SVE) metric that provides a bounded, interpretable measure of vaccine effect ranging from $-1$ (maximal harm) to 1 (maximal protection), and developing inferential methods including profile likelihood and delta method CIs that have not previously been studied for this class of parameters.

The outline of the remainder of this paper is as follows. Section 2 compares the proposed SVE estimand to the traditional VE parameter. Section 3 considers inference about the SVE estimand. Section 4 examines operating characteristics of the inferential methods through simulation. Section 5 illustrates the approach using data from a large HIV vaccine trial (rgp120 HIV Vaccine Study Group, 2005), demonstrating the interpretational advantages of SVE over traditional VE. Section 6 describes R code for estimating SVE and corresponding CIs.

## 2 Symmetric Vaccine Efficacy

VE is the standard measure used to quantify the effect of a vaccine (World Health Organization, 2025). In practice, VE is often defined as one minus a relative measure of risk, such as the relative risk or hazard ratio. For example, in a trial setting with infection probabilities $p_1$ if vaccinated and $p_0$ if not vaccinated, the VE parameter is often defined as


(1) \begin{eqnarray*}
\mathrm{VE}=1-\frac{p_1}{p_0},
\end{eqnarray*}

with $\mathrm{VE}=1$ corresponding to complete protection (ie, no risk of infection if vaccinated) and $\mathrm{VE}=0$ corresponding to the null of no effect (ie, equal risk regardless of whether vaccinated) (Halloran et al., 1991). The VE parameter as defined in Equation (1) is bounded above by 1 but unbounded below, making it inherently asymmetric.

Alternatively, a SVE can be defined as


(2) \begin{eqnarray*}
\mathrm{SVE}=\frac{p_0-p_1}{\max (p_0, p_1)}.
\end{eqnarray*}

By construction, SVE is bounded between $-1$ and 1, and has a straightforward interpretation as follows.

1. When $p_0> p_1$, $\mathrm{SVE}=1-p_1/p_0$ will be greater than 0 and equal to VE. Thus, in this case, SVE has the same interpretation as the traditional VE metric, that is, the proportional reduction in risk due to vaccination. SVE values closer to 1 indicate a stronger protective effect.
2. When $p_1> p_0$, $\mathrm{SVE}=p_0/p_1-1$ will be less than zero. Negative SVE captures the proportional decrease in risk from not being vaccinated, with values closer to $-1$ corresponding to more possible harm due to vaccination on the same scale as the protective effect of vaccination.

To illustrate the contrast between traditional VE and the proposed SVE, Figure 1 shows L’Abbe plots (L’Abbé et al., 1987) of infection probabilities if vaccinated versus if not vaccinated. In these plots, iso-effect lines correspond to pairs of infection probabilities $(p_0,p_1)$ with the same effect size. For example, the 45-degree line indicates pairs of equal infection probabilities (ie, the vaccine has no effect such that infection risk is the same in both groups). Iso-effect lines for traditional VE shown in Figure 1 a correspond to $p_1 = (1-\mathrm{VE})p_0$, whereas the iso-effect lines for SVE shown in Figure 1 b are given by $p_1 =(1-\mathrm{SVE})p_0$ if $\mathrm{SVE}\ge 0$ and $p_1=p_0/(1+\mathrm{SVE})$ if $\mathrm{SVE}< 0$. In contrast to VE, the SVE iso-effect lines are symmetric about the 45-degree line, with SVE bounded between $-1$ and 1 for all possible values of $(p_0,p_1)$. That is, SVE is a balanced metric where both protection and harm are represented on the same bounded scale, highlighting its interpretability advantages.

**Figure 1:**
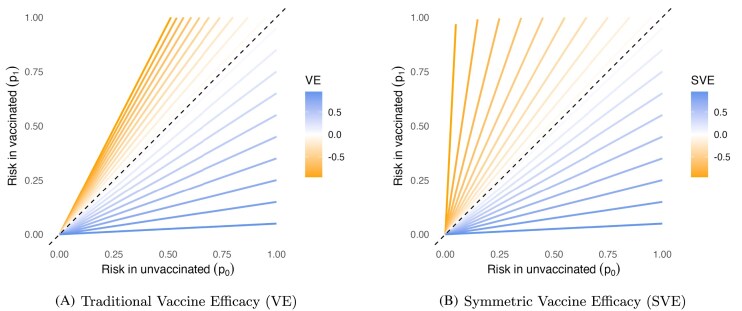
L’Abbe plots illustrating iso-effect lines for (A) traditional VE and (B) SVE. Each line represents a fixed effect size, with 20 evenly spaced values from $-0.95$ to 0.95 ranging from strong harm to strong protection represented by the line color. The dashed diagonal line at $y = x$ indicates equal risk in vaccinated and unvaccinated groups. For traditional VE, the iso-effect lines above this diagonal (negative effect sizes) appear compressed compared to those below it (positive effect sizes), illustrating the metric’s inherent asymmetry. By contrast, the symmetric SVE iso-effect lines remain bounded and mirror protective and harmful effects around the diagonal, providing a more interpretable measure of VE.

## 3 Inference for Sve

Consider a randomized trial with perfect compliance and no loss to follow-up. Let $X_0\sim \mathrm{Binomial}(n_0,p_0)$ and $X_1 \sim \mathrm{Binomial}(n_1,p_1)$ denote the numbers of infections observed in the unvaccinated and vaccinated groups, respectively, where $n_0$ and $n_1$ are the group sample sizes (Halloran et al., 1991). Assume $p_0,p_1 \in (0,1)$. Denote the empirical risks in the two groups by $\hat{p}_0=X_0/n_0$ and $\hat{p}_1=X_1/n_1$, which are unbiased estimators of the true infection risks $p_0$ and $p_1$. Define the plug-in estimator of SVE to be $\widehat{\mathrm{SVE}}=(\hat{p}_0-\hat{p}_1)/\max (\hat{p}_0,\hat{p}_1)$, which is obtained by substituting the empirical risks for the true risks in Equation (2). By the law of large numbers and continuous mapping theorem, it follows that $\widehat{\mathrm{SVE}}$ is a consistent estimator of SVE as $n_0,n_1 \rightarrow \infty$. Like other ratio estimators (Jewell, 1986), $\widehat{\mathrm{SVE}}$ will in general exhibit finite-sample bias. In Web Appendix B.1, a bias-corrected estimator is proposed which may be preferred in small sample size settings. In Sections 3.1 and 3.2, CIs for SVE using the profile likelihood and delta method are presented.

### 3.1 Profile likelihood CIs

A CI for SVE can be obtained by inverting a likelihood ratio test, as follows. First note that $\hat{p}_0$ and $\hat{p}_1$ are the unconstrained maximum likelihood estimators (MLEs) of the log-likelihood


(3) \begin{eqnarray*}
\ell (p_0,p_1)=\sum _{j=0}^1\lbrace X_j\log (p_j)+(n_j-X_j)\log (1-p_j)\rbrace .
\end{eqnarray*}

Likelihood-based inference of the SVE parameter can be achieved via the profile likelihood, wherein for any value of SVE the likelihood is maximized over $p_0$ and $p_1$, subject to the functional constraints implied by the value of SVE. In particular, if $\mathrm{SVE}\ge 0$, then the constraint is $p_1=p_0(1-\mathrm{SVE})$. On the other hand, if $\mathrm{SVE}< 0$, then the constraint is $p_0=p_1(1+\mathrm{SVE})$. Therefore, the profile log-likelihood for SVE is


(4) \begin{eqnarray*}
\ell _p(\mathrm{SVE})= \left\lbrace \begin{array}{@{}l@{\quad }l@{}}\displaystyle \sup _{p_0\in (0,1)}\ell (p_0, p_0(1-\mathrm{SVE})) & \text{if SVE}\in [0,1),\\\displaystyle \sup _{p_1\in (0,1)}\ell (p_1(1+\mathrm{SVE}),p_1) &\text{if SVE}\in (-1,0). \end{array}\right.
\end{eqnarray*}

The two cases in Equation (4) each entail a one-dimensional maximization over either $p_0$ or $p_1$ which can be easily solved using standard one-dimensional numerical optimization algorithms.

Consider testing the null hypothesis $H_0:\mathrm{SVE}=s$ for some $s \in (-1,1)$. Define the likelihood ratio statistic $\Lambda (s)=2\lbrace \ell (\hat{p}_0,\hat{p}_1)-\ell _p(s)\rbrace$, where $\ell (\hat{p}_0,\hat{p}_1)$ is the maximum of the unconstrained log-likelihood (3), and $\ell _p(s)$ is the profile log-likelihood (4) evaluated at $\mathrm{SVE}=s$. Larger values of $\Lambda (s)$ provide stronger evidence against $H_0:\mathrm{SVE}=s$.

Under standard regularity conditions as $n_0,n_1\rightarrow \infty$, $\Lambda (s)$ converges in distribution to a $\chi _1^2$ under the null. Using this asymptotic approximation, the likelihood ratio test can be inverted in the usual fashion to construct a CI for SVE. Specifically, the $(1-\alpha )\%$ profile likelihood CI for SVE is $\lbrace s \in (-1,1):\Lambda (s)\le \chi ^2_{1,1-\alpha }\rbrace$, where $\chi ^2_{1,1-\alpha }$ denotes a $(1-\alpha )$-quantile of a $\chi ^2_1$ distribution.

### 3.2 Wald CIs

For large samples, Wald CIs provide a computationally efficient alternative to the profile likelihood approach. Let $n=n_0+n_1$ be the total trial size and assume $n_0/n\rightarrow \lambda \in (0,1)$ as $n \rightarrow \infty$. Then, under standard regularity conditions and assuming independence between groups, by the bivariate central limit theorem


\begin{eqnarray*}
&& \sqrt{n} {\begin{pmatrix}\hat{p}_0-p_0\\\hat{p}_1-p_1 \end{pmatrix}}\xrightarrow {d} \boldsymbol{\mathcal {N}}\left(\mathbf {0},\boldsymbol{\Sigma }\right),\\&& \text{where }\boldsymbol{\Sigma }= {\begin{pmatrix}p_0(1-p_0)/\lambda & 0\\0 & p_1(1-p_1)/(1-\lambda ) \end{pmatrix}}.
\end{eqnarray*}

The multivariate delta method in turn implies that $\widehat{\mathrm{SVE}}$ is also asymptotically normal. In particular, let $g(p_0,p_1)=\mathrm{SVE}=(p_0-p_1)/\max (p_0,p_1)$ such that $g(\hat{p}_0,\hat{p}_1)=\widehat{\mathrm{SVE}}$. Note that the gradient $\boldsymbol{\nabla }g$ exists and is continuous throughout the interior of the parameter space, despite the maximum operator in the denominator (see Web Appendix A). Therefore, $\sqrt{n}(\widehat{\mathrm{SVE}}-\mathrm{SVE}) \xrightarrow {d} \boldsymbol{\mathcal {N}}(0,\boldsymbol{\nabla }g^\top \boldsymbol{\Sigma }\boldsymbol{\nabla }g)$. The asymptotic variance can be consistently estimated by replacing $p_0$ and $p_1$ with $\hat{p}_0$ and $\hat{p}_1$, and $\lambda$ with $\hat{\lambda }= n_1/n$. Letting $\hat{\sigma }_0^2=\hat{p}_0(1-\hat{p}_0)/n_0$ and $\hat{\sigma }_1^2=\hat{p}_1(1-\hat{p}_1)/n_1$, it follows (see Web Appendix A for details) that in large samples, the variance of $\widehat{\mathrm{SVE}}$ can be approximated by


(5) \begin{eqnarray*}
\widehat{\mathrm{Var}}(\widehat{\mathrm{SVE}})=\frac{\hat{p}_1^2\hat{\sigma }^2_0+\hat{p}^2_0\hat{\sigma }^2_1}{\lbrace \max (\hat{p}_0, \hat{p}_1)\rbrace ^4},
\end{eqnarray*}

which simplifies to $(\hat{\sigma }^2_0+\hat{\sigma }^2_1)/\hat{p}^2$ when $\hat{p}_0=\hat{p}_1=\hat{p}$. Thus a $100(1-\alpha )\%$ Wald-type CI for SVE is $\widehat{\mathrm{SVE}} \pm z_{1-\alpha /2} \widehat{\mathrm{Var}}(\widehat{\mathrm{SVE}})^{1/2}$, where $z_{1-\alpha /2}$ is the ($1-\alpha /2$) quantile of the standard normal distribution. These CIs are asymptotically valid but can include values outside the parameter space $[-1,1]$ when the SVE estimate lies near the boundaries or in settings where there is considerable uncertainty (eg, in small samples).

Wald CIs may also be computed on a transformed scale to ensure that the lower and upper endpoints of the CI remain within the parameter space $[-1,1]$ after back-transformation. For example, the SVE estimator can be transformed using the inverse hyperbolic tangent $\hat{u}=\tanh ^{-1}(\widehat{\mathrm{SVE}})=0.5\log \lbrace (1+\widehat{\mathrm{SVE}})/(1-\widehat{\mathrm{SVE}})\rbrace$, where $\hat{u}$ can take on any real value in $(-\infty , \infty )$ (Fisher, 1921; Hotelling, 1953; van der Vaart, 1998). By the delta method, $\mathrm{Var}(\widehat{u}) \approx \mathrm{Var}(\widehat{\mathrm{SVE}})/(1-\mathrm{SVE}^2)^2$ for which an estimator $\widehat{\mathrm{Var}}(\hat{u})$ can be constructed by replacing SVE with $\widehat{\mathrm{SVE}}$ and $\mathrm{Var}(\widehat{\mathrm{SVE}})$ with the variance estimator in Equation (5). The corresponding CI for SVE is obtained by back-transformation:


\begin{eqnarray*}
\left[\tanh \left(\widehat{u} -z_{1-\alpha /2}\sqrt{\widehat{\mathrm{Var}}(\widehat{u})}\right), \tanh \left(\widehat{u} +z_{1-\alpha /2}\sqrt{\widehat{\mathrm{Var}}(\widehat{u})}\right)\right].
\end{eqnarray*}

Hereafter, these are termed *tanh-Wald intervals*.

### 3.3 Extension to relative effect measures

In some settings, a vaccine’s effect may be estimated by $\widehat{\mathrm{VE}}=1-\hat{\theta }$ where $\hat{\theta }$ is an estimated rate ratio from a Poisson regression model or an estimated hazard ratio from a Cox proportional hazards model. The SVE framework extends naturally to any multiplicative relative effect measure $\theta$ by defining


(6) \begin{eqnarray*}
\mathrm{SVE}=\frac{1-\theta }{\max (1,\theta )},
\end{eqnarray*}

where $\theta =1$ corresponds to the vaccine having no effect, in which case SVE $=0$. When $\theta < 1$, the vaccine is protective and Equation (6) simplifies to $\mathrm{SVE} = 1 - \theta$. When $\theta > 1$, the vaccine is harmful and Equation (6) becomes $\mathrm{SVE} = (1 - \theta )/\theta$. In this setting, SVE can be estimating by replacing $\theta$ in Equation (6) with an estimator $\hat{\theta }$ obtained using standard likelihood-based procedures, for example by the MLE from a Poisson model or the maximum partial likelihood estimator from a Cox proportional hazards models. Let $\ell (\theta )$ denote the corresponding log-likelihood or partial log-likelihood, and let $\hat{\theta }$ be the unconstrained maximizer of $\ell (\theta )$.

CIs for SVE defined by Equation (6) can be constructed using profile likelihood or the delta method, similar to Sections 3.1 and 3.2. To construct profile likelihood CIs, for each candidate SVE value *s*, the profile likelihood $\ell _p(s)$ is obtained by maximizing $\ell (\theta )$ subject to $\mathrm{SVE}=s$. The resulting likelihood ratio statistic $\Lambda (s)=2\lbrace \ell (\hat{\theta })-\ell _p(s)\rbrace$ quantifies evidence against $H_0: \mathrm{SVE}=s$. Under standard regularity conditions as $n_0,n_1\rightarrow \infty$, $\Lambda (s)$ is asymptotically $\chi ^2_1$ under the null. The corresponding $(1-\alpha )$ profile likelihood CI again has the form $\lbrace s \in (-1,1):\Lambda (s)\le \chi ^2_{1,1-\alpha }\rbrace$.

Wald-type CIs may be obtained using the delta method applied to the log-scale relative effect. Let $\hat{\phi }= \log (\hat{\theta })$ with estimated variance $\widehat{\mathrm{Var}}(\hat{\phi })$. The variance of $\widehat{\mathrm{SVE}}$ can then be estimated by


(7) \begin{eqnarray*}
\widehat{\mathrm{Var}}(\widehat{\mathrm{SVE}}) = \frac{\widehat{\mathrm{Var}}(\hat{\phi })}{\lbrace \max (\hat{\theta }, \hat{\theta }^{-1})\rbrace ^{2}}.
\end{eqnarray*}

When $\hat{\theta }=1$, Equation (7) reduces to $\widehat{\mathrm{Var}}(\hat{\phi })$.

## 4 Operating Characteristics

This section presents results from Monte Carlo simulation studies of the SVE estimator and corresponding CIs described in Section 3. Bias, sampling variability, and CI coverage were examined across a range of infection probabilities and group sample sizes. Comparisons were also made to traditional VE-based inference in terms of CI width and type I error rate.

### 4.1 Simulation setup

In each simulated data set (replicate), the numbers of infected individuals in the vaccinated and unvaccinated groups were generated independently from binomial distributions with sample sizes $n_1$ and $n_0$ and infection probabilities $p_1$ and $p_0$, respectively. Underlying parameters were varied over a grid of values. Infection probabilities were set to $p_0,p_1\in \lbrace 0.1,0.2, \dots , 0.9\rbrace$ and sample sizes $n_0, n_1\in \lbrace 100,250,1000\rbrace$ were used.

For each setting, 10 000 replicates were generated. For each replicate, the empirical infection risks $\hat{p}_1$ and $\hat{p}_0$ were used to compute $\widehat{\mathrm{SVE}}$ along with profile likelihood, original-scale Wald, and tanh-Wald 95% CIs. Coverage probabilities were estimated by the proportions of CIs that contained the true SVE value. Type I error rates were estimated by the proportions of intervals that did not contain 0 when the true SVE value was 0, that is, when $p_0=p_1$.

### 4.2 Simulation results

Bias of $\widehat{\mathrm{SVE}}$ was negligible and the average variance estimates (5) closely approximated the empirical variance across the range of infection probabilities and sample sizes considered (see Web Figures 1 and 2). Figure 2 displays the empirical coverage probabilities for the profile likelihood, original-scale Wald, and tanh-Wald CIs as a function of true SVE, stratified by sample size. Across all scenarios, the profile likelihood intervals provided coverage probabilities closest to the nominal 95% level. For small sample sizes ($n_0=n_1=100$), the profile likelihood and tanh-Wald intervals maintained coverage closer to the nominal 95% level across the full range of SVE values, while the original-scale Wald intervals tended to undercover near extremes (ie, when the true SVE is close to $-1$ or 1). As sample sizes increased, coverage for all three CIs stabilized around 95%.

**Figure 2:**
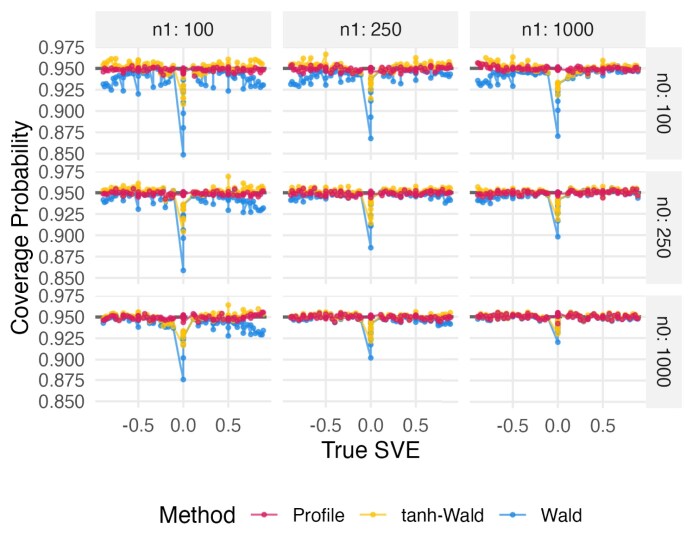
Empirical coverage probabilities of 95% CIs of SVE as a function of true SVE values. Three types of CIs were considered: a profile likelihood interval (Profile), a Wald interval on the original SVE scale (Wald), and a back-transformed tanh-Wald interval (tanh-Wald). Each panel corresponds to a combination of sample sizes for the vaccinated and unvaccinated groups with columns corresponding to values of $n_1 \in \lbrace 100, 250, 1000\rbrace$ and rows to $n_0 \in \lbrace 100, 250, 1000\rbrace$. Coverage across the full range of SVE values is close to the nominal level for the profile likelihood and tanh-Wald intervals, with modest undercoverage for the original-scale Wald intervals at smaller sample sizes. Larger sample sizes stabilize coverage for all methods.

The empirical type I error rates for the setting when $\mathrm{SVE}=0$ (the null case) are presented in Web Appendix B.4 (Web Figure 4). The type I error was well controlled when sample sizes were large and balanced, whereas smaller or imbalanced designs led to inflated type I errors for the Wald intervals. The empirical type I error was closest to the nominal 5% for the profile likelihood intervals, followed by the tanh-Wald intervals, and poorest for the original-scale Wald intervals.

### 4.3 Comparison to traditional VE

Figure 3 compares SVE 95% CIs (tanh-Wald) to traditional VE 95% CIs for 25 randomly selected simulated data sets when $p_0=p_1=0.1$ and $n_0=n_1=100$. The SVE intervals are symmetrically distributed around the null of 0. However, traditional VE estimates yield highly asymmetric intervals, with the lower bound often extending far below zero and potentially giving the false impression that an ineffective vaccine is actually harmful.

**Figure 3:**
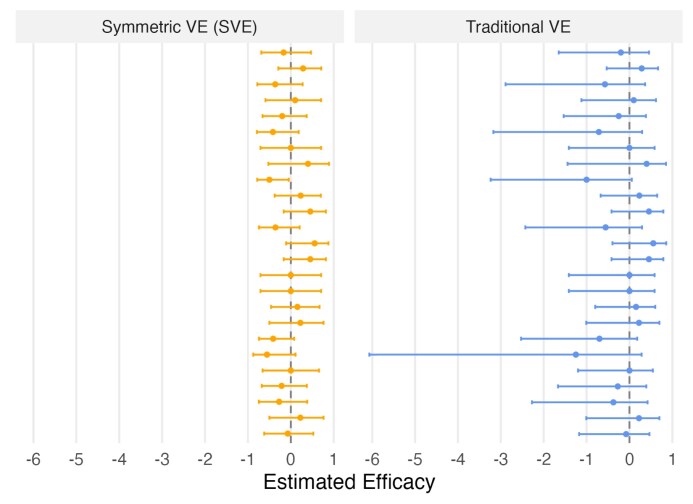
Forest plot of vaccine effect estimates and CIs from 25 simulated data sets under the null (no vaccine effect) with infection risk $p_0=p_1=0.1$ and sample sizes $n_0=n_1=100$. Each point represents the estimated effect with error bars representing 95% CIs for SVE and traditional VE. SVE intervals (calculated as tanh-Wald intervals) are guaranteed to be contained $[-1,1]$ and symmetrically distributed around the null, whereas traditional VE intervals are highly asymmetric, with lower bounds extending far below zero.

Additional comparison of the widths of 95% CIs for SVE and traditional VE for simulations where $n_0=n_1={10,00}$ are presented in Web Appendix B.3 (Web Figure 3). When the vaccine was protective (ie, $p_1 < p_0$), estimates of SVE and VE tended to be similar and not surprisingly the corresponding CIs had similar average width. However, when the vaccine was harmful (ie, $p_1 > p_0$), CIs for traditional VE became extremely wide, while those for SVE remained stable. CIs for traditional VE were sometimes >80 times as wide as those for SVE in the extreme cases (ie, when $p_1$ was much higher than $p_0$).

## 5 Application to an Hiv Vaccine Trial

VAX004 was a placebo-controlled phase 3 trial of a recombinant glycoprotein 120 (rgp120) vaccine designed to prevent HIV-1 infection (rgp120 HIV Vaccine Study Group, 2005). The trial randomized ${5403}$ participants (2:1 allocation, $n_1={3598}$, $n_0={1805}$) to receive seven injections of the rgp120 vaccine or placebo over 30 months, with HIV-1 seroconversion as the primary endpoint. The analysis presented here utilizes the number of HIV-1 infections in the vaccine and placebo groups from Table 3 of rgp120 HIV Vaccine Study Group (2005), with VE estimated as one minus the ratio of the proportion infected in the two trial arms. Due to the unavailability of individual-level data, the hazard ratio from a Cox proportional hazards model, as reported in the original study, was not estimated.

VE was estimated overall and across several subgroups (eg, by age, race, and baseline behavioral risk score), as in the original study. Overall and subgroup-level infection proportions as well as VE and SVE estimates are given in Web Appendix C (Web Table 1). Figure 4 presents a forest plot of $\widehat{\mathrm{VE}}$ and $\widehat{\mathrm{SVE}}$ with 95% CIs across the subgroups. The 95% CIs for the SVE were calculated using the profile likelihood method.

**Figure 4:**
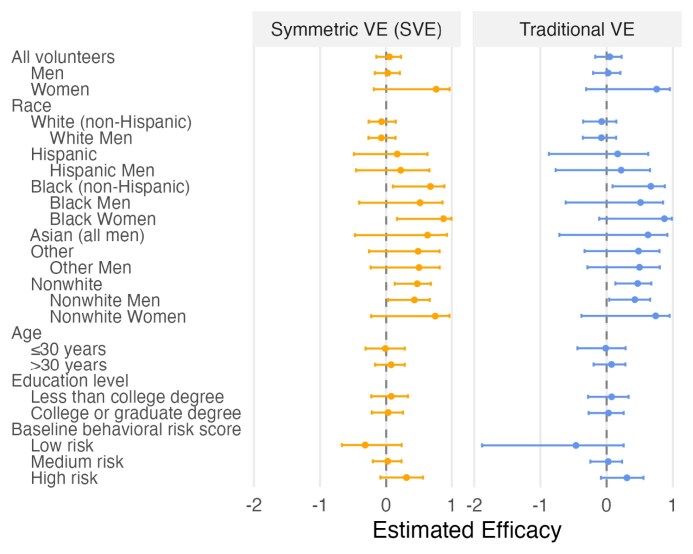
Forest plot comparing traditional VE and SVE against HIV-1 infection in the rgp120 Trial. Each point represents the estimated efficacy with error bars representing 95% CIs. SVE CIs were calculated using the profile likelihood method. Data from rgp120 HIV Vaccine Study Group (2005).

The results in Figure 4 illustrate two key advantages of SVE over traditional VE. First, $\widehat{\mathrm{SVE}}$ and the corresponding profile likelihood CI are bounded within $[-1,1]$, which precludes extremely negative point estimates or CI endpoints. This is particularly important in smaller subgroups where there may be substantial uncertainty. For example, in the low baseline behavioral risk score subgroup, the VE 95% CI extends far below zero ($-1.88$ to 0.26), whereas the SVE 95% CI only spans $-0.67$ to 0.23.

Second, as a symmetric measure, SVE is easier to interpret than traditional VE. A negative SVE value indicates a protective effect of the placebo relative to the vaccine on the same scale for which a positive SVE indicates a protective effect of the vaccine relative to the placebo. For example, in the low baseline behavioral risk score subgroup, the lower bound $-0.67$ of the SVE CI indicates that the placebo group had 67% reduced infection risk relative to the vaccine group, while the CI upper bound 0.23 indicates that the vaccine group had 23% reduced infection risk relative to the placebo group. For this particular subgroup, there is considerable uncertainty due to the small sample sizes and relatively low proportion infected (2.9% in the vaccinated group versus 1.8% in the unvaccinated group). As a result, the CI for traditional VE occupies a disproportionately large span in the forest plot in Figure 4, which may give the misleading impression that the vaccine is likely to be harmful in this subgroup. To the contrary, these results plausibly mirror the simulated results in Figure 3, where the true effect is null rather than harmful. On the other hand, SVE provides a more balanced depiction of the possible vaccine effects across the subgroups. The bounded CIs have more reasonable width, better reflecting the underlying uncertainty without potentially implying exaggerated harm. That said, SVE and VE are distinct parameters, and therefore CIs for SVE and VE are not directly comparable. The narrower SVE intervals in this application reflect the bounded nature of the parameter itself, that is, SVE is constrained by construction, whereas VE is unbounded below. As a result, SVE CIs necessarily fall within [−1, 1], unlike VE CIs.

## 6 Software Implementation in R

To facilitate estimation of SVE and it associated uncertainty, the R package sve was developed. The package provides functions for estimating SVE, the estimator’s variance, and CIs as described in Section 3. The sve package can be installed from GitHub using the devtools package:

# install.packages(“devtools”)

devtools{::}install_github(“LucyMcGowan/sve”)

The core function est_sve() outputs an SVE estimate with a corresponding CI based on inputs consisting of the observed number of infections and sample sizes for the vaccinated and unvaccinated groups. For example, suppose in a two-arm placebo-controlled randomized vaccine trial that the observed proportion of infected participants is $\hat{p}_0 = 0.1$ in the placebo arm and $\hat{p}_1 = 0.05$ in the vaccine arm, with $n_0 = n_1 = 1000$ participants in each arm. This code

library(sve)

est_sve(x0 = 100, x1 = 50, n0 = 1000, n1 =1000)

produces the following output:

#> estimate lower upper level method

#> 1 0.50 0.31 0.64 0.95 Profile

Here, the estimated SVE is 0.50, with 95% CI $(0.31, 0.64)$ calculated using the profile likelihood method. The profile likelihood method is applied by default (method = ”profile”); to use tanh-Wald intervals or original-scale Wald intervals instead, the user may select method = ”tanh-wald” or method = ”wald”.

The sve_from_model() function computes estimates and CIs of SVE from estimates of a relative effect measure (eg, hazard ratio from a Cox model, or rate ratio from Poisson regression) as described in Section 3.3. For example, a Cox-model based estimate of SVE and corresponding profile likelihood 95% CI can be computed for the simulated trial dataset (sim_trial_data) included in the sve package via the following code:

library(survival)

fit <- coxph(Surv(time, status) vaccination + age + baseline_risk,

data = sim_trial_data)

sve_from_model(model = fit, data = sim_trial _data, effect_name = “vaccination”)

#> estimate lower upper level method

#> 1 0.68 0.59 0.75 0.95 Profile

## 7 Discussion

Traditional VE is widely used and easily interpretable when the vaccine is protective and uncertainty is small (eg, in large trials). However, VE’s inherent asymmetry can create difficulties in interpretability and communication. In particular, VE is unbounded below which can lead to highly asymmetric CIs in practice. Resulting extreme negative values of the VE can misleadingly suggest that a vaccine is harmful when the true effect may simply be small or null.

The SVE proposed in this paper addresses these limitations by defining efficacy on a bounded symmetric scale from $-1$ (maximally harmful) to 1 (maximally protective). This scale provides a natural and interpretable measure that treats protection and harm equivalently. Standard large sample frequentist inference is considered in the randomized trial setting where a binary outcome (eg, infection) is observed for all trial participants. SVE can easily be adapted to accommodate other relative effect measures. For example, in vaccine trials where time to infection is the primary outcome, VE is commonly estimated as one minus the hazard ratio from a Cox proportional hazards model, which can be used in place of the relative risk in the SVE estimator, as described in Section 3.3. To support broad use of SVE, open-source software is provided for implementing the SVE estimator in R, as described in Section 6.

In Monte Carlo simulations, the SVE estimator and corresponding profile likelihood and tanh-Wald CIs performed well across a wide range of settings. Under the null (when the vaccine has no effect), CIs for the SVE remain symmetric around the null, while those for traditional VE can be severely asymmetric. This feature of SVE may be particularly important for subgroup analyses and early-stage studies where small numbers of infections may lead to misleadingly wide or negative lower bounds for traditional VE.

The practical advantages of SVE are highlighted in the HIV vaccine trial analysis presented in Section 5. In one subgroup, the traditional VE 95% CI had a strongly negative lower bound, visually dominating forest plots and potentially giving the false impression that the vaccine was harmful. In contrast, SVE produced a bounded interval for all subgroups while accurately conveying the uncertainty in these estimates.

Utilization of SVE may improve communication about vaccine effectiveness to nontechnical audiences, policymakers, and the public, by whom negative VE estimates can be easily misinterpreted and potentially cause disproportionate concern. Relative effect measures are inherently challenging to interpret (Brown, 2022; Dupont and Plummer, 1996), and the additional asymmetry of traditional VE can exacerbate misunderstandings. A natural question is whether SVE is more interpretable than VE for different audiences, including public health officials, clinicians, and patients.

Traditional VE can be challenging to interpret because harm and benefit are measured on different scales: $(-\infty ,0)$ for harm and (0,1] for benefit. Consequently, positive values of VE are not directly comparable to negative values of VE. For example, VE = 40% and VE = $-40$% are not effects of comparable magnitude, despite having the same absolute value. Likewise, if a VE 95% CI equals (–200%, 67%), then correct interpretation of the upper and lower bounds requires recognition that a 67% reduction in risk and a 200% increase in risk are in fact effects of comparable magnitude (but opposite direction).

SVE, on the other hand, does not require such nuanced understanding because benefit and harm are measured on the same scale. Nevertheless, one potential concern is that SVE’s changing denominator adds some complexity to interpretation. In particular, whereas positive values of SVE indicate the relative reduction in risk due to vaccination, negative values of SVE quantify the relative reduction in risk due to *not* receiving the vaccine.

Ultimately, whether reporting VE or SVE improves understanding of vaccine effects for different groups of individuals (eg, subject-matter experts, public health officials, and so forth) is an important empirical question. Further study of SVE in the broader context of health communication and evidence-based medicine is needed.

## Supplementary Material

ujag141_Supplemental_Files Web Appendices, Tables, and Figures referenced in Sections 3−5 are available with this paper at the Biometrics website on Oxford Academic.

## Data Availability

Code to replicate all analyses, tables, and figures can be found in the Supplementary Materials. Additionally, they can be found on GitHub here: https://github.com/LucyMcGowan/symmetric-ve. The data referenced in Section 5 were obtained from Table 3 of rgp120 HIV Vaccine Study Group (2005) and have also been made available in the Supplementary Materials and in our GitHub repository.
