# Supplementary material for: Symmetric vaccine efficacy
Source: Biometrics. 2026 Aug 11;82(3):ujag141. doi: 10.1093/biomtc/ujag141 (PMC13459110; doi:10.1093/biomtc/ujag141)
Supplement: Supplementary file 1 [file ujag141_supplemental_files.zip › supplement.pdf]

Supplementary Materials for “Symmetric Vaccine Efficacy”

by Lucy D’Agostino McGowan, Sarah C. Lotspeich, and Michael G. Hudgens

Web Appendix A. Delta Method and Variance Estimation

This appendix provides details regarding the delta method derivation of the asymptotic variance of $\widehat{\text{SVE}}$. In the main text, the multivariate delta method is used to show that $\widehat{\text{SVE}}$ is asymptotically normal. The delta method requires the gradient ∇g of $g(p_0, p_1) = (p_0 - p_1)/\max(p_0, p_1)$ exists and is continuous throughout the interior of the parameter space. To show this, note $g(p_0, p_1)$ is defined piecewise according to whether $p_0 > p_1$ or $p_0 < p_1$. To establish differentiability, consider the partial derivatives within each region. When $p_0 > p_1$

$$\frac{\partial g}{\partial p_0} = \frac{p_1}{p_0^2}, \quad \frac{\partial g}{\partial p_1} = -\frac{1}{p_0},$$

whereas for $p_0 < p_1$

$$\frac{\partial g}{\partial p_0} = \frac{1}{p_1}, \quad \frac{\partial g}{\partial p_1} = -\frac{p_0}{p_1^2}.$$

Next note the partial derivatives also exist at points (p, p) along the boundary between the regions; in particular,

$$\partial g/\partial p_0 = \lim_{h \to 0^+} \frac{g(p+h, p) - g(p, p)}{h} = \lim_{h \to 0^-} \frac{g(p+h, p) - g(p, p)}{h} = \frac{1}{p}$$

and

$$\partial g/\partial p_1 = \lim_{h \to 0^+} \frac{g(p, p+h) - g(p, p)}{h} = \lim_{h \to 0^-} \frac{g(p, p+h) - g(p, p)}{h} = -\frac{1}{p}.$$

Finally, note the partial derivatives are continuous, including at points (p, p), i.e.,

$$\lim_{(p_0, p_1) \to (p, p)} \frac{\partial g}{\partial p_0} = \frac{1}{p}, \quad \lim_{(p_0, p_1) \to (p, p)} \frac{\partial g}{\partial p_1} = -\frac{1}{p}.$$

Thus, the gradient of $g(p_0, p_1)$ exists and is continuous throughout the interior of the parameter space, including along the diagonal where $p_0 = p_1$. It then follows from the delta method that in large samples

$$\text{Var}(\widehat{\text{SVE}}) \approx \frac{1}{n} \nabla g^\top \boldsymbol{\Sigma} \nabla g = \begin{cases} \dfrac{p_1^2 \sigma_0^2 + p_0^2 \sigma_1^2}{p_0^4} & \text{if } p_0 > p_1 \\[2ex] \dfrac{p_1^2 \sigma_0^2 + p_0^2 \sigma_1^2}{p_1^4} & \text{if } p_1 \geqslant p_0 \end{cases} \tag{S.1}$$

where $\sigma_j^2 = p_j(1-p_j)/n_j$ for $j = 0, 1$. This variance can be consistently estimated by the plug-in estimator $\widehat{\mathrm{Var}}(\widehat{\mathrm{SVE}})$, defined by replacing p_j and σ_j in (S.1) with $\hat{p}_j$ and $\hat{\sigma}_j^2$ for $j = 0, 1$.

Web Appendix B.

Web Appendix B.1 *Estimator Finite-sample Bias*

The estimator $\widehat{\mathrm{SVE}}$ is consistent for SVE, but exhibits finite-sample bias, a known issue for ratio estimators (Jewell, 1986). When $p_0 \neq p_1$, a second-order Taylor expansion gives the following approximations to the bias. When $p_0 > p_1$,

$$\mathrm{Bias}(\widehat{\mathrm{SVE}}) = \mathbb{E}(\widehat{\mathrm{SVE}}) - \mathrm{SVE} \approx -p_1(1-p_0)/(n_0 p_0^2).$$

Note

$$\left| -\frac{p_1(1-p_0)}{n_0 p_0^2} \right| \leqslant \frac{p_1(1-p_0)}{n_0 p_0^2} \leqslant \frac{1-p_0}{n_0 p_0} \leqslant \frac{1}{n_0 p_0}$$

Thus the bias will tend to be small except in smaller trials where $n_0 p_0$, the expected number of events in the control arm, is small. Likewise, when $p_1 > p_0$,

$$\mathrm{Bias}(\widehat{\mathrm{SVE}}) = \mathbb{E}(\widehat{\mathrm{SVE}}) - \mathrm{SVE} \approx p_0(1-p_1)/(n_1 p_1^2),$$

which is bounded above by $1/(n_1 p_1)$. The results above suggest the finite-sample bias of $\widehat{\mathrm{SVE}}$ might be partially mitigated by using the bias-corrected SVE estimator

$$\widehat{\mathrm{SVE}}_{\mathrm{BC}} = \begin{cases} \widehat{\mathrm{SVE}} + \frac{\hat{p}_1(1-\hat{p}_0)}{n_0 \hat{p}_0^2} & \text{if } \hat{p}_0 > \hat{p}_1, \\ \widehat{\mathrm{SVE}} - \frac{\hat{p}_0(1-\hat{p}_1)}{n_1 \hat{p}_1^2} & \text{if } \hat{p}_1 > \hat{p}_0, \\ \widehat{\mathrm{SVE}} & \text{if } \hat{p}_0 = \hat{p}_1. \end{cases} \tag{S.2}$$

The bias corrected estimator subtracts an estimator of the bias from the original SVE estimator, using plug-in estimates $\hat{p}_0$ and $\hat{p}_1$ in place of p_0 and p_1. The correction is asymptotically negligible (vanishing at rate $O(n^{-1})$) but may reduce bias in finite samples.

Web Figure 1 shows the bias $\widehat{\mathrm{SVE}}$ and $\widehat{\mathrm{SVE}}_{\mathrm{BC}}$ across the simulation scenarios. The empirical

bias of $\widehat{\text{SVE}}$ shown in Web Figure 1a is small or negligible across all scenarios. The finite-sample bias is greatest when p_0 and p_1 are small and the corresponding sample size is small. Web Figure 1b shows the bias of $\widehat{\text{SVE}}_{\text{BC}}$, demonstrating that the bias correction effectively reduces the finite-sample bias in scenarios with small sample sizes and low event probabilities.

(a) $\widehat{\text{SVE}}$

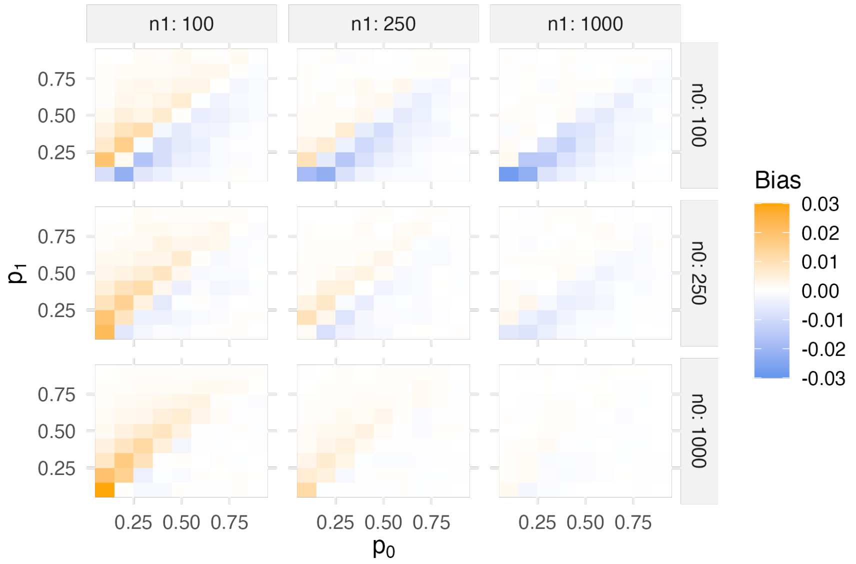

(b) $\widehat{\text{SVE}}_{\text{BC}}$

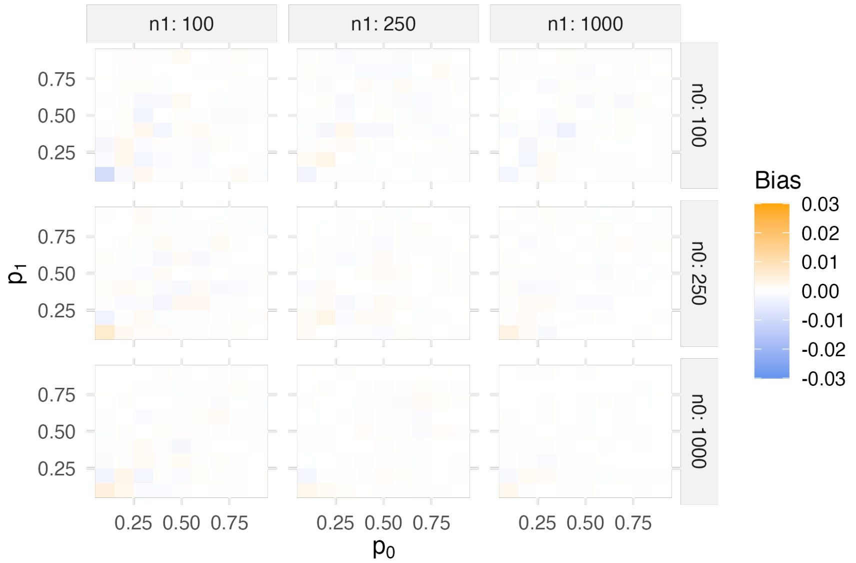

Web Figure 1: Bias heatmap for the SVE estimator **(a)** without the bias correction and **(b)** with the bias correction. Each cell represents the average bias ($\widehat{\text{SVE}} - \text{SVE}$ or $\widehat{\text{SVE}}_{\text{BC}} - \text{SVE}$) over 10,000 simulations for a given combination of risk if unvaccinated (p_0), risk if vaccinated (p_1) and sample sizes (n_0 and n_1). Both estimators exhibit relatively minimal bias across all scenarios, with the bias-corrected SVE estimator $\widehat{\text{SVE}}_{\text{BC}}$ having slightly reduced bias compared to the uncorrected estimator $\widehat{\text{SVE}}$, particularly in scenarios with smaller sample sizes and lower risks.

Web Appendix B.2 *Standard Error Ratio*

Web Figure 2 shows the ratio of averaged estimated standard error $\widehat{\text{Var}}(\widehat{\text{SVE}})^{1/2}$ to the empirical standard error (SE) of $\widehat{\text{SVE}}$ from the simulation study. The ratios are close to 1 across all scenarios, indicating accurate variance estimation.

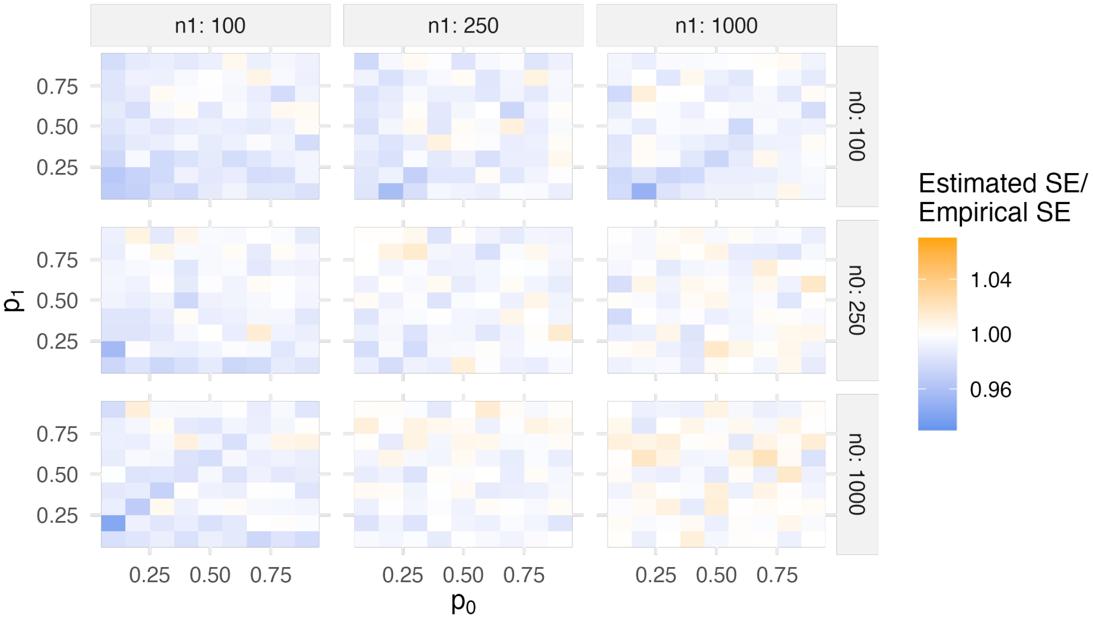

Web Figure 2: Ratio of averaged estimated standard error $\widehat{\text{Var}}(\widehat{\text{SVE}})^{1/2}$ to the empirical standard error (SE) of $\widehat{\text{SVE}}$ from the simulation study. Values near 1 (white) indicate accurate variance estimation. Orange indicates overestimation of the standard error, while blue indicates underestimation.

Web Appendix B.3 *Comparison of SVE and VE Confidence Interval Widths*

Web Figure 3 shows the ratio of 95% CI widths for traditional VE versus SVE for simulations where $n_0 = n_1 = 1000$. When the vaccine is protective (i.e., $p_1 < p_0$), estimates of SVE and VE tend to be similar with CIs of similar average width. However, when the vaccine is harmful (i.e., $p_1 > p_0$), confidence intervals for traditional VE become extremely wide, while those for SVE remain stable. Confidence intervals for traditional VE were more than 80 times as wide as those for SVE in the extreme case when p_1 was much higher than p_0.

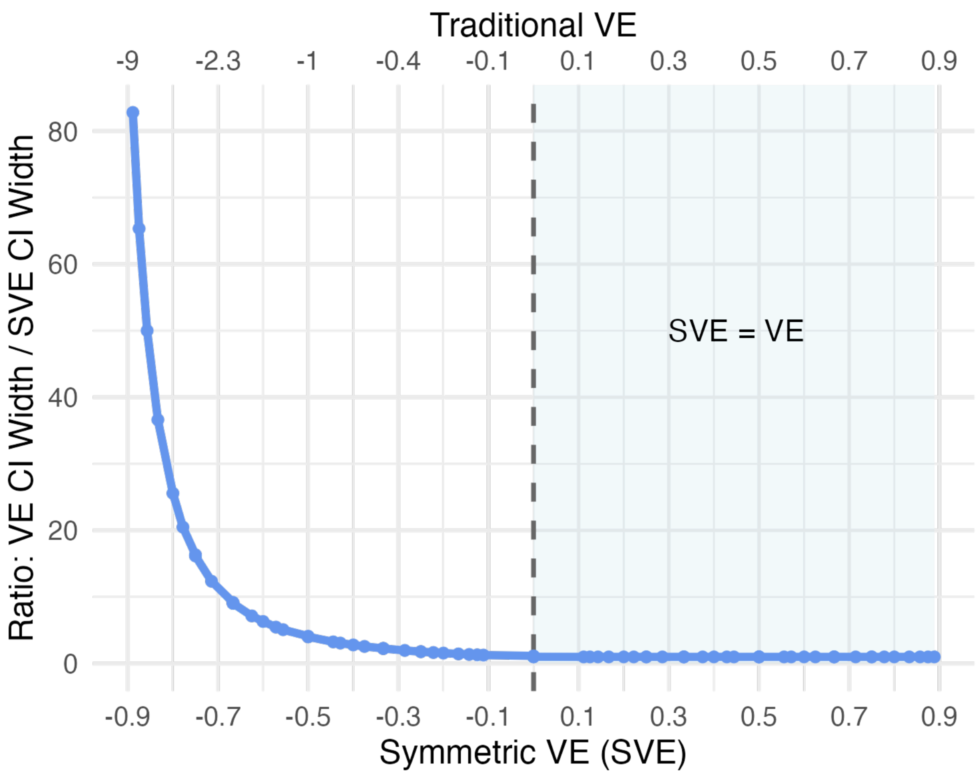

Web Figure 3: The ratio of 95% confidence interval widths for traditional vaccine efficacy (VE) to symmetric vaccine efficacy (SVE). The bottom horizontal axis shows values of SVE, and the corresponding values of traditional VE are shown on the top horizontal axis. The shaded region indicates where VE = SVE, corresponding to positive efficacy. When efficacy is negative (unshaded region), the traditional VE confidence intervals can become extremely wide relative to SVE confidence intervals. The vertical dashed line represents where VE and SVE are both equal to 0.

Web Appendix B.4 *Type I Error*

Web Figure 4 presents empirical type I error rates under the null (SVE = 0). Type I error control was strongest for the profile likelihood intervals, followed by the tanh-Wald intervals, and poorest for the original-scale Wald intervals.

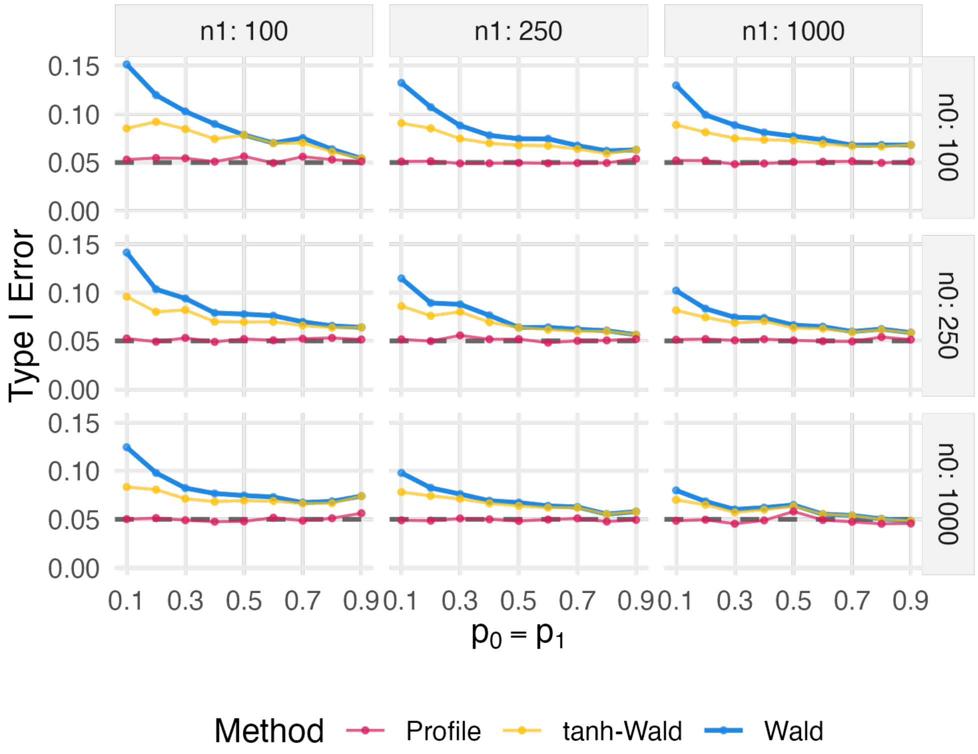

Web Figure 4: Type I error rates under the null hypothesis (vaccine has no effect) across varying shared infection risks ($p_0 = p_1$) and sample sizes (n_0, n_1). Three types of confidence intervals were considered: a profile likelihood interval (Profile), a Wald interval on the original SVE scale (Wald), and a back-transformed tanh-Wald interval (tanh-Wald). Each panel displays the empirical type I error as a function of the infection risk, with columns corresponding to the vaccinated group sample size n_1 and rows to the unvaccinated group sample size n_0. The dashed horizontal line marks the nominal level of 0.05. When sample sizes are large and balanced (e.g., $n_0 = n_1 = 1{,}000$), type I error is relatively well controlled across all infection risks. However, type I error is more variable in smaller sample sizes, particularly at small infection risks (e.g., $p_0 = p_1 = 0.1$), where noticeable inflation is observed for both Wald-type methods. The profile likelihood interval maintains nominal type I error across all scenarios.

Web Appendix C. Application to an HIV Vaccine Trial

Overall and subgroup-level empirical HIV-1 infection risks are shown in Web Table 1, as well as VE and SVE estimates with corresponding 95% confidence intervals (CIs). The CIs for SVE were obtained using the profile likelihood method.

Web Table 1: Estimated risk of HIV-1 infection (number of infections/number of participants) in the vaccine and placebo groups, with estimates and 95% confidence intervals (CIs) of traditional vaccine efficacy (VE) and symmetric vaccine efficacy (SVE)

Category	Vaccine	Placebo	VE (95% CI)	SVE (95% CI)
All volunteers	241/3598 (0.07)	127/1805 (0.07)	0.05 (-0.17 to 0.23)	0.05 (-0.15 to 0.22)
Men	239/3391 (0.07)	123/1704 (0.07)	0.02 (-0.20 to 0.21)	0.02 (-0.17 to 0.21)
Women	2/207 (0.01)	4/101 (0.04)	0.76 (-0.31 to 0.95)	0.76 (-0.19 to 0.97)
Race				
White (non-Hispanic)	211/2994 (0.07)	98/1495 (0.07)	-0.08 (-0.36 to 0.15)	-0.07 (-0.26 to 0.14)
Men	211/2930 (0.07)	98/1468 (0.07)	-0.08 (-0.36 to 0.14)	-0.07 (-0.27 to 0.14)
Hispanic	14/239 (0.06)	9/128 (0.07)	0.17 (-0.87 to 0.63)	0.17 (-0.49 to 0.62)
Men	13/211 (0.06)	9/114 (0.08)	0.22 (-0.77 to 0.66)	0.22 (-0.46 to 0.65)
Black (non-Hispanic)	6/233 (0.03)	9/116 (0.08)	0.67 (0.09 to 0.88)	0.67 (0.10 to 0.89)
Men	5/121 (0.04)	5/59 (0.08)	0.51 (-0.62 to 0.85)	0.51 (-0.41 to 0.86)
Women	1/112 (0.01)	4/57 (0.07)	0.87 (-0.11 to 0.99)	0.87 (0.16 to 0.99)
Asian (all men)	3/56 (0.05)	3/21 (0.14)	0.62 (-0.71 to 0.92)	0.62 (-0.47 to 0.93)
Other	7/76 (0.09)	8/45 (0.18)	0.48 (-0.33 to 0.80)	0.48 (-0.26 to 0.81)
Men	7/73 (0.10)	8/42 (0.19)	0.50 (-0.29 to 0.80)	0.50 (-0.23 to 0.81)
Nonwhite	30/604 (0.05)	29/310 (0.09)	0.47 (0.13 to 0.68)	0.47 (0.13 to 0.68)
Men	28/461 (0.06)	25/236 (0.11)	0.43 (0.04 to 0.66)	0.43 (0.03 to 0.66)
Women	2/143 (0.01)	4/74 (0.05)	0.74 (-0.38 to 0.95)	0.74 (-0.23 to 0.96)
Age				
$\leqslant$ 30 years	84/971 (0.09)	43/504 (0.09)	-0.01 (-0.44 to 0.29)	-0.01 (-0.31 to 0.28)
$>$ 30 years	157/2627 (0.06)	84/1301 (0.06)	0.07 (-0.20 to 0.28)	0.07 (-0.17 to 0.28)
Education level				
Less than college degree	95/1409 (0.07)	52/713 (0.07)	0.08 (-0.28 to 0.33)	0.08 (-0.22 to 0.33)
College or graduate degree	146/2188 (0.07)	75/1092 (0.07)	0.03 (-0.27 to 0.26)	0.03 (-0.22 to 0.25)
Baseline behavioral risk score				
Low risk	32/1211 (0.03)	11/609 (0.02)	-0.46 (-1.88 to 0.26)	-0.32 (-0.67 to 0.23)
Medium risk	177/2229 (0.08)	90/1107 (0.08)	0.02 (-0.25 to 0.23)	0.02 (-0.20 to 0.23)
High risk	32/158 (0.20)	26/89 (0.29)	0.31 (-0.08 to 0.56)	0.31 (-0.09 to 0.56)

References

Jewell, N. P. (1986). On the bias of commonly used measures of association for 2 x 2 tables. *Biometrics* **42,** 351–358.
